# The Effects of Visual Control and Distance in Modulating Peripersonal Spatial Representation

**DOI:** 10.1371/journal.pone.0059460

**Published:** 2013-03-15

**Authors:** Chiara Renzi, Emiliano Ricciardi, Daniela Bonino, Giacomo Handjaras, Tomaso Vecchi, Pietro Pietrini

**Affiliations:** 1 Laboratory of Clinical Biochemistry and Molecular Biology, University of Pisa Medical School, Pisa, Italy; 2 Deptartment of Brain and Behavioral Sciences, University of Pavia, Pavia, Italy; 3 Fondazione Toscana Gabriele Monasterio CNR-Regione Toscana, Pisa, Italy; 4 Brain Connectivity Center, IRCCS Mondino, Pavia, Italy; 5 Clinical Psychology Branch, Pisa University Hospital, Pisa, Italy; University of Milan, Italy

## Abstract

In the presence of vision, finalized motor acts can trigger spatial remapping, i.e., reference frames transformations to allow for a better interaction with targets. However, it is yet unclear how the peripersonal space is encoded and remapped depending on the availability of visual feedback and on the target position within the individual’s reachable space, and which cerebral areas subserve such processes. Here, functional magnetic resonance imaging (fMRI) was used to examine neural activity while healthy young participants performed reach-to-grasp movements with and without visual feedback and at different distances of the target from the effector (near to the hand–about 15 cm from the starting position–vs. far from the hand–about 30 cm from the starting position). Brain response in the superior parietal lobule bilaterally, in the right dorsal premotor cortex, and in the anterior part of the right inferior parietal lobule was significantly greater during visually-guided grasping of targets located at the far distance compared to grasping of targets located near to the hand. In the absence of visual feedback, the inferior parietal lobule exhibited a greater activity during grasping of targets at the near compared to the far distance. Results suggest that in the presence of visual feedback, a visuo-motor circuit integrates visuo-motor information when targets are located farther away. Conversely in the absence of visual feedback, encoding of space may demand multisensory remapping processes, even in the case of more proximal targets.

## Introduction

Peripersonal space is a preferential sector of space, immediately surrounding the body, which exhibits a high degree of multisensory interactions [1]–[3]. Within such space visual, auditory and tactile information is integrated, likely to allow for a better guidance of voluntary, as well as defensive actions directed towards objects (e.g., [4]–[7]). In fact, it has been hypothesized that the functional significance of peripersonal space resides in expressing sensory (i.e., localization of an object in space) and motor responses (i.e., directing an action towards an object or performing a movement to defend ourselves from an approaching object) within a common reference frame [8], [9]. On the other hand, the reachable space is functionally defined as the portion of the environment reached by extending the arm without moving the trunk. Despite being proximal to the body, this sector is encoded as extrapersonal space [1].

Features and boundaries of peripersonal space can extend to the reachable space through reference frames transformations often referred to as ‘spatial remapping’ (see e.g., [10]), depending on the available sensory information and action performance. For instance, behavioral studies investigating movement planning in relation to peripersonal space demonstrated that object affordances trigger actions depending on their reachability in space [11], and that the intention to perform an action on a target makes the latter being perceived as closer compared to when no action is planned [12]. Accordingly, recent studies suggested that grasping and reaching motor acts may trigger remapping of peripersonal space in early phases of action, but such remapping can interact with the availability of visual feedback [13]–[15]. In fact, the sensory modality providing the main stream of information during an action (e.g., visual rather than proprioceptive only) may affect the way space is perceived and processed [16], [17]. However, which cerebral areas are involved in spatial remapping, and to what extent neural activity in these regions is modulated by the type of sensory feedback available, remains to be fully understood.

Among the action repertoire, grasping has been extensively characterized. This motor act is controlled by a cortical network that partially overlaps with areas devoted to spatial representation (e.g., [18]–[22]). Furthermore, despite being relevant for modulating the transport component of the motor act, visual feedback is not a mandatory requisite for the effective performance of grasping (e.g., [23]–[28]).

In the present study, we assessed the neural correlates of grasping actions in relation to sensory feedback availability and peripersonal space coding. Specifically, we sought to determine (i) whether and how the sensory modality providing the main input for action (i.e., visually- vs. proprioceptively-guided actions) modulates neural activity in action- or space-related areas; (ii) the influence of sensory feedback on the neural correlates of peripersonal space representation by manipulating target distance (near to vs. far from the hand) while performing an action.

Functional magnetic resonance imaging (fMRI) was used to measure brain activity in healthy individuals who were asked to perform different grips while changing the availability of visual feedback and the distance of the target. Of note, as compared to previous imaging works investigating multisensory representation of the space around the hand (e.g., [29]–[31]) or distance encoding during action performance [32], the novelty of the present study is the manipulation of both sensory feedback and distance during a motor act.

On the basis of the neurophysiological findings described above, we predicted that distance encoding would interact with the type of sensory feedback available by modulating neural activity in the fronto-parietal cortical network related to the representation of peripersonal space during action performance.

## Methods

### Participants

Fifteen healthy right-handed participants (10 M, mean age±S.D. = 28±9 years, with normal or corrected to normal visual acuity) were enrolled in the study and took part in a fMRI scanning session in which the grasping task was performed with proprioceptive feedback only (see Experimental Procedure). Nine of these volunteers (7 M, mean age ± S.D. = 24±3 years) subsequently performed a separate session with visual feedback on a different day (see Experimental Procedure). All participants received a clinical examination, including routine blood tests and a brain structural MRI scan to exclude any disorder that could affect brain functions and metabolism. None of the volunteers was taking any medication for at least four weeks prior to the study.

### Ethics Statement

Informed written consent was obtained prior to enrollment in the study and after the study procedures and risks involved had been explained to each participant. The study was conducted under a protocol approved by the University of Pisa Ethical Committee (protocol n. 1616/2003), and was developed in accordance with the Declaration of Helsinki (2008).

### Stimuli and Stimulation Apparatus

A small (diameter = 4 cm) and a large (diameter = 10 cm) polystyrene sphere, fixed on a wooden pole (length = 80 cm) were used as stimuli. During the fMRI scanning, poles with the polystyrene balls leaned on a polystyrene support fixed on the MRI table and were held fix in place by an experimenter. The volunteers had their forearms comfortably resting on a pad that was placed on the participants' abdomen as a benchmark for the starting and return point of the right hand. Arms were restrained at the elbow with cushions to minimize consequent head movements.

### Experimental Procedure

Prior to the fMRI scanning session, participants underwent training in order to familiarize with the task. Training blocks (including six grasping trials each) were repeated until volunteers reached 100% accuracy. A minimum of two, and up to eight blocks, were performed. During the fMRI exam, additional practice trials were performed to enable participants to learn the position of the stimuli before each scanning session.

A fast event-related design with variable Inter Stimulus Intervals (ISI), ranging between 3.5 and 8 s with an exponential probability, was applied. Each run included four blocks of stimulation, which lasted 70 s and included six grasping trials and a period of rest after each block. For each participant in the visual feedback condition (see below) and four participants in the proprioceptive feedback condition (see below), six runs were acquired for a total of 144 grasping trials. For 11 participants in the proprioceptive feedback condition, eight runs were acquired for a total of 192 grasping trials.

Before each block, participants were orally instructed about the size of the stimulus they were going to deal with (the word “*piccolo*” – the Italian for *small* – for the 4 cm sphere and the word “*grande*” – the Italian for *large* – for the 10 cm sphere). During fMRI, participants were asked to reach and grasp with their right dominant hand either using a precision grip (PG) or a whole hand grasp (WHG). A high tone (l = 250 ms, frequency - f − = 850 Hz, sawtooth waveform) was the cue corresponding to PG, while a low tone cue (l = 250 ms, f = 210 Hz, sawtooth waveform) corresponded to WHG. Sounds were delivered through pneumatic MRI-compatible headphones that were controlled via Presentation (Neurobehavioral Systems Inc., Albany, CA, USA). Even though, as a first step of analyses, the present work did not address the modulation of the neural correlates of grasping-specific factors, the use of different grips with different target sizes was preferred over the use of a single grip repeated over the runs to introduce a certain degree of task variability and to limit the distinct influence of these grasping-specific factors on the results.

Grasping conditions were randomized within the individual blocks and balanced within the single runs, while the size of the stimuli was randomized and balanced across runs but was kept constant within a single block of stimulation.

**Figure 1:**
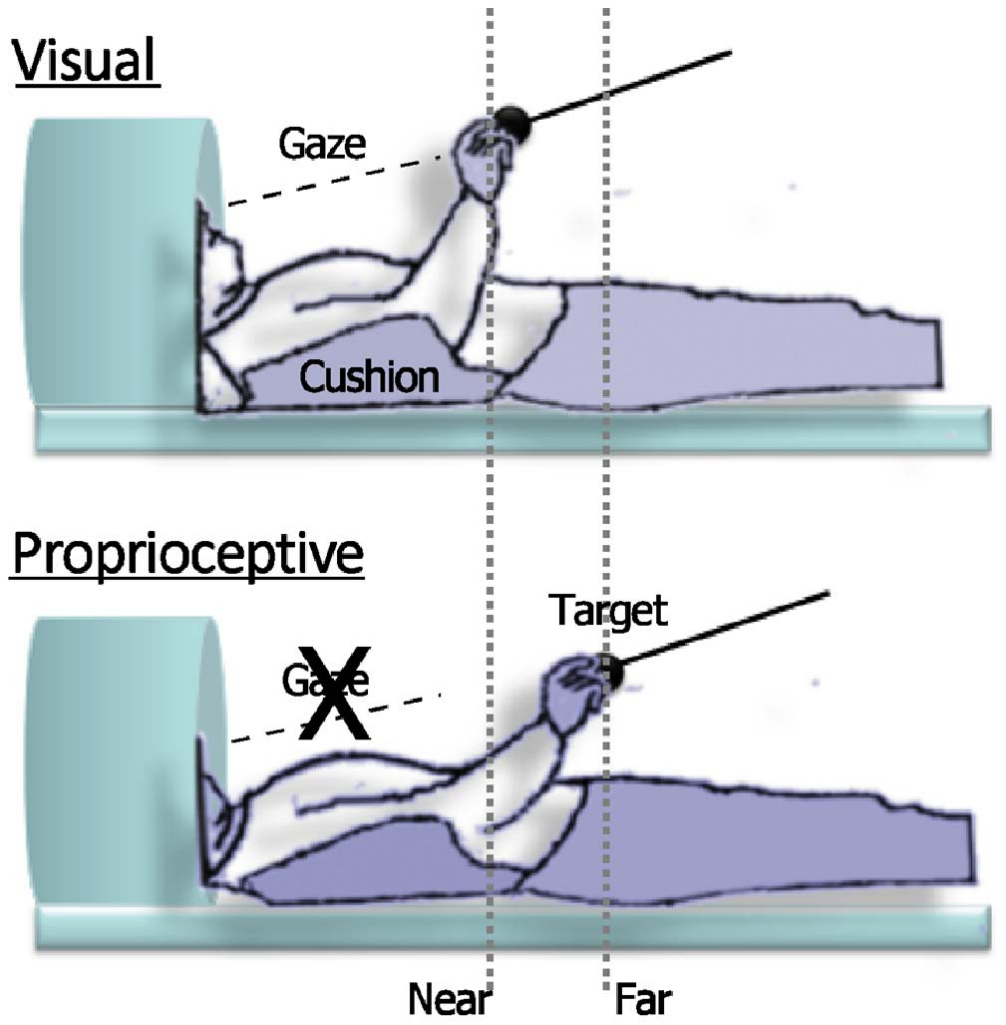
Schematic view of the experimental setup. Illustration of participants and target position (near or far from the hand) in the scanner during the visual (panel above) and proprioceptive (panel below) feedback conditions.

The spheres to be grasped were placed at two different distances: ‘near’ to the hand (15 cm from the pad, thus easily reachable), or ‘far’ from the hand (approximately 30 cm from the pad, and in order to reach almost the maximal arm extension without moving the trunk or the head; see Figure 1). Thus, the target positions were defined both in an absolute (i.e., the distance between the starting pad positioned on the abdomen from the target) and in a relative manner. In fact, the far distance corresponded to a specific functional benchmark (i.e., the maximal arm extension) relatively to the body of the participant. To compensate for differences in arm length while keeping constant the covered distance, the location of the starting pad on the abdomen was adjusted for each participant in order to comply with these constraints. Distances were kept constant within runs.

Errors in execution (e.g., wrong sound-movement association, misreaching, anticipation of movement) were recorded by the experimenter and the corresponding trials were then removed from the analysis.

In the proprioceptive feedback condition, volunteers performed the task with their eyes closed. Nine of the volunteers also performed a session with visual feedback on a different day after the proprioceptive feedback condition session. For this session, participants had their head tilted at an angle of approximately 30° in order to let them see the stimuli directly, without the use of projecting mirrors, to avoid any spatial separation between visual and proprioceptive cues. Stimuli were introduced at the same time of the auditory cue for the size and removed within 5 seconds after the last trial of the block. Task instructions and procedures were the same as the proprioceptive feedback session. Each session lasted approximately 1.5 hours.

### Image Acquisition

Gradient echo echoplanar (GRE-EPI) images were acquired with a 1.5 Tesla scanner (Signa General Electric, Milwaukee, WI, USA). A scan cycle (repetition time = 2500 ms) was composed of 27 axial slices (4 mm thickness, field of view = 24 cm, echo time = 40 ms, flip angle = 90°, image in-plane resolution = 96×96 pixels) collected during grasping motor acts.

For each participant, six to eight time series, consisting of 114 volumes, were registered. High resolution T1-weighted spoiled gradient recall images were obtained for each participant to provide detailed brain anatomy.

### Data Analysis

The AFNI package was used to analyze functional imaging data (http://afni.nimh.nih.gov/afni [33]). After reconstruction of raw data, slice acquisition times were aligned (3dTshift), linear and quadratic trend were removed (3dDetrend). Volumes from all runs were registered to the volume collected nearest in time to the acquisition of the high-resolution anatomical scan (3dvolreg). Spatial smoothing was performed with an isotropic Gaussian filter (sigma = 3.5). Blood oxygenation level dependent (BOLD) response was calculated in percent signal change with respect to volumes of rest.

Individual time points relative to those motor acts that were classified as errors (see Methods section) were censored (0.8% of the overall time points), and thus not included in the analysis. A multiple linear regression (3dDeconvolve) was used to model each regressor of interest using a block response function. Each regressor consisted in the task type (PG, WHG) by size (small, large) by distance (near, far) condition for a total of eight regressors of interest for each feedback condition. The six movement parameters derived from the volume registration, the polynomial trend regressors, ventricular signal averaged from a ventricular ROI, and signal averaged from a white matter ROI were included in multiple regression as nuisance regressors to reduce physiological cardiac and respiratory pulsatility and residual noise related to grasping residual movement artifacts (see [34], [35]). The impulse response functions were obtained from each regressor of interest for each volunteer, and the mean of the first six timepoints (corresponding to 12.5 s, and able to fully model the hemodynamic response) was calculated and used in the group-level analysis.

Single subject data were then registered in standard space according to the Talairach and Tournoux atlas (1988) and resampled to 1 mm^3^. At the second level of analysis, a three-way mixed-model group ANOVA with Object Distance (near, far) as a within subjects factor, Sensory Feedback (proprioceptive, visual) as a between subjects factor, Participants as a random factor and BOLD percent signal change as dependent variable was conducted to identify areas significantly involved with the availability of the visual feedback, with the distance of the target, and in the interaction between the object’s location and the sensory modality providing the main input for action guidance. The correction for multiple comparisons across the whole brain was conducted using MonteCarlo simulations run via AlphaSim in AFNI with a voxel-wise threshold of 0.001, that resulted in a minimum cluster of 964 voxel, with a cluster connection radius of 1 mm for a corrected *p* value of 0.001 at cluster level.

## Results

**Figure 2:**
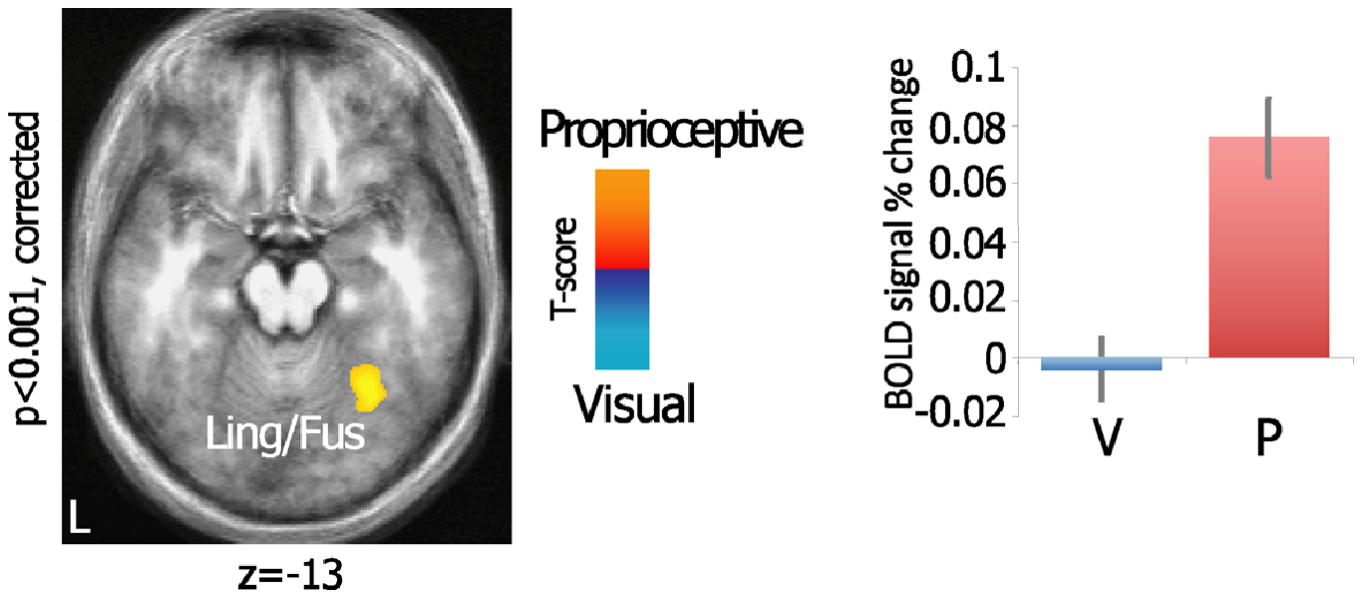
Visual feedback availability during grasping modulates lingual/fusiform cortex. Axial view of the statistical map shows the direct comparison between visual and proprioceptive feedback conditions within the ANOVA, projected onto a group-averaged structural template. Bar graphs illustrate the mean ± SE of the relative BOLD signal intensity (% change) across the experimental conditions for each of the significant clusters. V *–* visual; P *–* proprioceptive; Ling/Fus *–* lingual and fusiform gyri.

**Figure 3:**
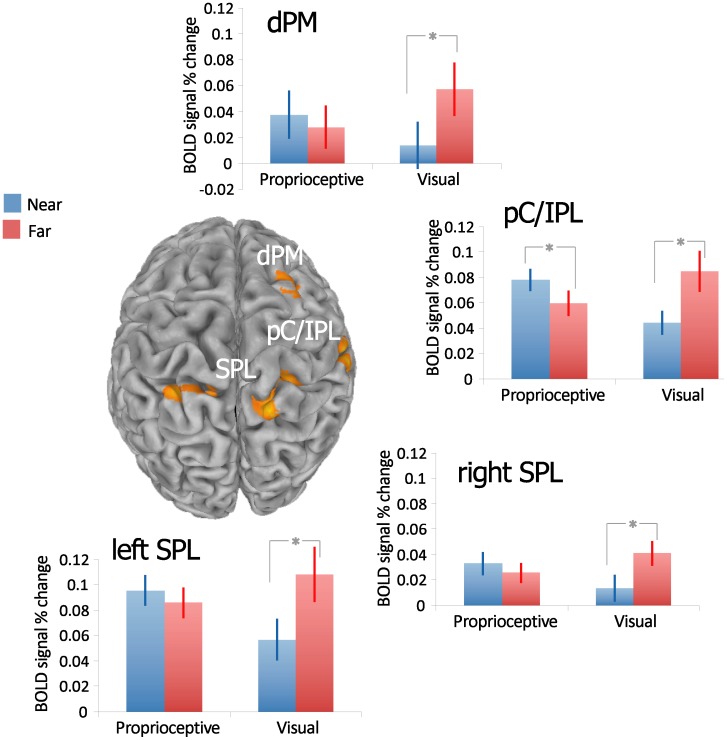
Grasping-related areas are modulated by the sensory modality x object distance interaction. Statistical map shows the Sensory Modality x Object Distance interaction within the ANOVA. Spatially normalized activations are projected onto a single-subject brain template in Talairach space. Bar graphs illustrate the mean ± SE of the relative BOLD signal intensity (% change) across the experimental conditions for each of the significant clusters. Significance for distance effect in each sensory modality conditions was reported for T-test p-value<0.05. dPM *–* dorsal premotor; pC/IPL *–* post central cortex/inferior parietal lobule; SPL *–* superior parietal lobule.

All the participants were able to perform the task correctly (mean error rate±sd for the proprioceptive and the visual feedback condition respectively: 0.77%±0.87%, 2.24%±2.01%; independent samples t-test: t_(21)_ = 2.10, *p* = 0.048). To investigate the effects of the availability of visual feedback, we directly compared the proprioceptive and the visual feedback conditions within the ANOVA (equivalent to a two-tailed independent samples t-test). One right-sided cluster encompassing part of the lingual gyrus superiorly and the posterior portion of the fusiform gyrus (including the parahippocampal area; BA 19/36/37) was significantly more activated in the proprioceptive as compared to the visual feedback condition (F_(1,22) = _14.38, *p* = 0.001; see Table 1 and Figure 2). No areas were found more activated in the opposite contrast. Furthermore, the main effect of Object Distance in the ANOVA resulted in no significant differential clusters. The Sensory Feedback x Object Distance interaction (see Table 1 and Figure 3) found significant clusters in the right dorsal premotor cortex (dPMC; BA 6) and in the parietal areas: specifically, a more anterior cluster, confined between the right postcentral gyrus (BA2), the inferior parietal lobule (IPL; BA40) and the supramarginal gyrus (SMG), and a bilateral posterior cluster in the superior parietal lobule (SPL; BA7).

**Table 1:** Cluster sizes and Talairach coordinates for significant brain regions from the three-way mixed-model group ANOVA with Object Distance as within subjects factor, Sensory Feedback as between subjects factor, Participants as random factor and the BOLD percent signal change as dependent variable.

Area	Hemisphere	Cluster size (in voxels)	CM x	CM y	CM z	Peak x	Peak y		Peak z	
***Sensory Feedback***										
Ling/Fus	L	2,709*	−29	−56	−12	−34		−59		−18
***Sensory Feedback x Object Distance***										
SPL	R	3,396*	+16.1	−53.5	+56.6	+7		−63		+62
pC/IPL	R	2,036*	+51.9	−31.2	+43.2	+49		−31		+51
SPL	L	1,589*	−20.3	−52.4	+56.7	−13		−50		+66
dPMC	R	1,375*	+24.5	−3.1	+61.6	+29		−5		+63

Note: CM – center of mass; Ling/Fus – lingual and fusiform gyri; SPL – superior parietal lobule; pC/IPL – post central gyrus/inferior parietal lobule; dPMC – dorsal premotor cortex; *p<0.001.

To analyze the pattern of activation of those areas showing a significant interaction, the average of time series for each cluster was calculated and two-tailed paired t-tests, comparing the near and far distance within the visual and proprioceptive feedback conditions were performed (see Figure 3). When visual feedback was available, the far distance resulted in a significantly higher coefficient compared to the near distance (right SPL: t_(8)_ = 4.110, *p* = 0.006; left SPL: t_(8)_ = 4.101, *p* = 0.006; right dPM: t_(8)_ = 6.664, *p*<0.001; right postcentral/IPL: t_(8)_ = 3.605, *p* = 0.014, Bonferroni correction) in all four clusters. In the absence of visual feedback, the postcentral/IPL cluster showed that the near distance had a significantly higher activity compared to the far distance (t_(14)_ = −3.499, *p* = 0.008, Bonferroni correction), while the SPL only showed a trend in this direction (right SPL: t_(14)_ = −1.793, *p* = 0.095; left SPL: t_(14)_ = −1.779, *p* = 0.097, uncorrected).

## Discussion

In this study, participants were asked to perform reach-to-grasp movements during two different sensory feedback (presence vs. absence of visual feedback, thus visually- vs. proprioceptively-guided movements) and distance (near to the hand vs. far from the hand) conditions.

In the first place, we examined separately the role of sensory feedback and distance during the task. Results showed that the sensory modality which guided the action did not modulate significantly areas in the dorsal stream, but rather an area in the ventral stream (see ‘Visual feedback availability’ subsection), supposed to be related to the spatial encoding of the object to grasp. Furthermore, no areas were found active in the encoding of distance across both sensory feedback conditions.

In the second place, the role of the visually-based control in the representation of peripersonal space for action was examined (see ‘Interaction of visual feedback with distance encoding’ subsection). In brief, brain responses in areas linked to spatial representation such as premotor and parietal areas, were modulated during the interaction of sensory feedback and object distance.

### Visual Feedback Availability

The first aim of this study was to determine whether and how the absence of visual feedback would affect brain response during the performance of grasping acts. The left fusiform gyrus was significantly activated when participants performed grasping in the absence of any visual feedback as compared to grasping under visual feedback; in contrast no significant activations were detected in the opposite comparison.

As in the proprioceptive feedback condition participants learned the spatial location of the target by performing a number of grasping actions prior to scanning, it may be hypothesized that this could have resulted in an allocentric encoding of the target’s position. Consistently, Committeri and coworkers [36] found an activation of the same area in the left ventromedial occipital-temporal cortex while participants encoded tridimensional information in landmark-centered coordinates compared to object-centered and viewer-centered perspectives. Moreover, the involvement of a closely located area in the ventral stream has been reported during the encoding phase of an object to be successively grasped [37].

Convergent pieces of evidence suggest that online action control in peripersonal space is supported by both egocentric (e.g., vision of the moving effector) and allocentric (e.g., visual information surrounding the target) encoding (see e.g., [38]–[40]). In turn, grasping parameters such as grip aperture are modulated by the presentation of pictorial illusions, which is considered indicative of the use of allocentric information [39]–[41].

To some extent, it may be considered surprising to hypothesize that allocentric information is used in relation to the execution of grasping motor acts in the complete absence of visual feedback. However, as our participants were allowed to open their eyes between runs, it cannot be ruled out that they actually relied on visual, ‘off-line’ allocentric information stored in memory. Nevertheless, the hypothesis that the absence of visual feedback results in a greater weighting of allocentric information, in addition to the egocentric information, may as well explain the finding that no significant differences in brain response were found when performing visually-guided grasping compared to the sole proprioceptive control.

On the other hand, as participants always performed the proprioceptive feedback session prior to the visual feedback session, one could argue that participants had a greater familiarity with the task during the visual feedback session and that this may contribute to explain, at least in part, the lack of statistically significant activation in this condition, as compared to the proprioceptive feedback one. However, grasping is a highly ecological task performed dozen of times each day, and is therefore minimally affected by a single repetition of a task, though relatively specific. Furthermore, considered that the time between the two sessions was at least 1 month and that the error rate was higher in the visual compared to the proprioceptive feedback condition, it could be considered as unlikely that a ‘learning’ or ‘repetition’ effect may have affected the data in any way.

### Interaction of Visual Feedback with Distance Encoding

The second aim of the study was to investigate how visual feedback availability may influence the representation and the spatial remapping of peripersonal space during an action. In general, the lack of significant activations in areas related to spatial cognition for the main effect of object distance, in addition to the finding that such areas are modulated by the interaction between the actual position of a target and the sensory modality guiding the action, strongly suggests that, within the brain, the spatial encoding of a target to grasp does not strictly depend on its absolute distance. Rather it can be hypothesized that spatial representation is influenced by other factors, such as the sensory feedback available when an action is performed. In fact, the activity of fronto-parietal cortical areas, such as bilateral SPL, right dPMC and right postcentral/IPL, exhibited a differential modulation of distance encoding depending on the modality providing the main source of feedback. More specifically, the activity of dPMC and bilateral SPL was modulated by distance only in the presence of visual feedback (greater activation for far compared to near targets).

We attribute the modulation of the dPMC and the SPL to visually-based remapping processes that would reflect the integration of hand and target location when the target is not included (or is distantly located) in the peripersonal space, as in the far distance condition. To perform a movement toward an object correctly, both the target and the effector have to be encoded within the same reference frame [42], [43]. Indeed, the integration of the target location with the hand initial position during a reaching task is correlated to activity in these areas [44]. Consistently, previous functional imaging studies reported activations in premotor and parietal areas overlapping with those clusters that have been found in our investigation, thus suggesting that this network may be related to spatial anticipation [45] and process spatial aspects of visually-guided actions (e.g., [46]).

Relatively to our task, the right dPMC may integrate the motor representation with the visual information about the target to update effector configuration and orientation during object approach [47]. Furthermore, a recent study reported a bilateral activation in the SPL when participants acted towards visual targets in the far peripersonal space compared to the near space [32]. The area of the right SPL found here overlaps in its posterior portion with the one characterized and labeled in Experiment 1 by Cavina-Pratesi and colleagues as right SPL/Area 5 L [32].

The SPL is crucially involved in the estimate of initial hand position and the encoding of the reach vector in the planning and early phases of action. Interestingly, SPL activity in monkeys is modulated by visual information when performing reaching movements [48], [49]. Similarly, in humans, the activity in the posterior part of the intraparietal sulcus is not modulated by the presence of a near compared to a far object when visual feedback is not available [31]. In accordance with our results, the encoding of hand-related space in this area may be mainly dominated by visual information [31].

Taking all these considerations into account, the functional meaning of the hand-target integration process, that is subserved by the activity of dPMC and SPL, may be related to the encoding of both the hand and the target within the same visual reference frame [43], [50], [51].

Differently from other areas, the pC/IPL also showed a greater activity in relation to the near distance compared to the far one when solely proprioceptive feedback was available. We hypothesize that the activity of this cluster may be related to a multisensory remapping of peripersonal space. More specifically, in the presence of visual feedback, far targets would fall outside of the peripersonal space; in contrast, in the absence of visual feedback, the near distance may already be encoded outside of peripersonal space and, thus, action performance would trigger remapping processes to integrate hand and target position. Studies in patients indicated that even if action programming can trigger *per se* peripersonal (with reaching) vs. extrapersonal (with pointing) space coding, the availability of visual feedback does play a role in spatial remapping [15]. When vision of a tool that is used to perform an action is prevented, an extrapersonal space representation is elicited in the near space [15]. Such pieces of evidence may explain the response of the pC/IPL cluster. The right IPL, in fact, is involved in personal/peripersonal space representation [52].

Moreover the activity in the aIPS is sensitive to the proprioceptive input to determine hand location and define the space near the hand. Therefore, aIPS has been hypothesized to represent the space near the hand by employing both somatosensory and visual hand coordinates [31]. Consistent with our hypothesis, primate cells recording and brain functional studies have shown an association between enhanced activity in the intraparietal sulcus and tools use that extended action space [53], [54]. However, due to intrinsic time resolution limitations of BOLD signal in fMRI, in our study it is not possible to determine whether the hypothesized remapping processes actually take place all-in-once e.g., at the action onset or throughout the time an action is carried on. To conclude, the differential activation pattern for near and far distances when performing grasping actions during the two different visual feedback conditions in the right pC/IPL may demand remapping processes. In turn, this reflects the modulation of peripersonal space boundaries given by the availability (or not) of visual feedback.

In sum, the findings of our study indicate that more dorsal cortical areas may be involved in spatial remapping processes that integrate visual information for action when an object is located farther away. On the other hand, the activity in a more ventral-anterior parietal area may reflect remapping processes for the boundaries of peripersonal space and thus encode peripersonal space flexibly depending on sensory feedback.
